# Domestic Medicine

**Published:** 1870-10

**Authors:** 


### Domestic Medicine.

### USEFUL RECIPES FOR THE HOUSEHOLD.

TREATMENT OF BLISTERS.—It is a common error that it is beneficial to break a blister in order to let out the water or blood which fills it. This water or blood is a healing substance, of a kind most appropriate for the parts where the skin is destroyed, and if the blister be allowed to dry, new skin forms more rapidly under it, and much pain is avoided. In exceptional cases, when the blister is very full of fluid, so that it causes much pain by its tension, a small portion of the fluid may be given a chance to escape, by the prick of a fine needle. A blister should also be covered up in some way to protect it from being ruptured by accident.

---

FEVER AND AGUE.—This distressing complaint so common in the south at this season of the year, can be broken up by the administration—every four hours—of the following mixture :—

Port Wine, 1 pint.
Pulv. Red Peruvian Bark, 1 ounce.
Quinine, 1½ drachms.
Aromatic Sul. Acid, 12 drops.

Mix, shake well, and take a teaspoonful every four hours. The remedy should be taken in teaspoonful doses every morning and evening for some time after the disease has subsided.

---

SALT RHEUM.—Take thirty grains of sulphate of copper (blue stone) and pulverize it to an impalpable powder. Add to this a half ounce of simple cerate, to be had at any drug store, and mix the two together thoroughly. Next wash the affected parts with castile soap and warm water, removing as many of the "scabs" as possible. Then apply the ointment to the raw surface. Follow this treatment every day until the parts are healed. In the meantime take :—

Iod. of Potassium, 30 grains.
Tincture of Iodine, ½ ounce.

Dissolve the Potassium in the Iodine, then add water four ounces. Take ten drops of the mixture three times per day, and you will entirely cure the difficulty.

---

ITCH.—Strip the person—wash in soap and water thoroughly—then sit upon a chair, throwing a thick blanket about the body, fastened under the chin. Then burn pulverized sulphur upon a hot ladle, under the blanket, until the body perspires very freely, after which dry the body and dress in linen. Repeat the application in a week if the patient does not fully recover.

---

INGROWING TOE-NAILS.—Procure a saturated solution of the persulphate of iron. Saturate a piece of cotton with it and insinuate it between the nail and the fungus flesh. Make this application morning and evening, wearing a loose shoe or slipper during the day.

---

WHITE OF EGG IN BURNS.—The white of an egg is a very efficatious remedy for burns. Apply it to the burned surface freely, with a feather. After a few applications the air is excluded and the pain ceases.

---

CROUP.—The season of year is approaching when croup may be expected among the children. Have the following medicine prepared and keep it in the house ready for use, when required.

Take :—
Tincture of Lobelia, 1 ounce.
Simple syrup, 3 ounces.
Mix.

Dose, half a teaspoonful to a teaspoonful every twenty minutes until relief is obtained.

---

ANOINTING IN DISEASE.—A celebrated English physician has been testing, with wonderful success, a very simple method of treating diseases of children, such as atrophy, bronchitis, convulsions, diarrhœa and febrile disturbances generally, as indeed all disturbances of childhood which are accompanied by an unnatural state of the skin.

The treatment consists simply in smearing with olive oil, the whole surface of the body from the crown of the head to the tips of the fingers and toes, the process being repeated every twelve, six, or even four hours, depending upon the urgency of the case. The child is kept in a long flannel gown or is wrapped in a small blanket after the anointing. The application of oil, the author writes, possesses the following immense advantages over the ordinary warm bath :—

"1. Skin action is more completely and permanently restored.

2. The danger of reaction is avoided, for there is no sudden change of temperature; and moreover the sheet of oil protects the surface from atmospheric influences.

3. It acts as a fuel-food, not only preventing waste of time, but actually increasing the bulk of the little patient.

4. It does not depress, but, on the contrary, appears to exhilerate.

It will scarcely be credited by many that the formidable affections above mentioned will frequently yield to this treatment, or at any rate show signs of abatement, from twenty minutes to twenty-four hours, but such is the case, though sometimes forty-eight or even seventy two hours will elapse before any decided signs of improvement occur."

---

FOR RINGWORM.—

Take:—

Washed sulphur, 22 grains.
Carbonate of Potash, 8 grains.
Lard, 1 ounce. Mix.

Apply to the parts morning and evening, continuing the application some time after the apparent cure, to prevent a return.

---

HOW TO CLEAN PAINT.—A very simple method for cleaning dirty paint is to provide a plate with some of the best whiting, and have ready some clean warm water and a piece of flannel, which dip into the water and squeeze nearly dry; then take as much whiting as will adhere to it, apply it to the painted surface, when a little rubbing will instantly remove any dirt or grease. After which wash the part well with clean water, rubbing it dry with a soft chamois. Paint thus cleaned looks as well as when first lain on, without any injury to the most delicate colors. It is far better than using soap, and does not require more than half the time and labor.

---

TO DESTROY FLIES.—The smoke of the dried leaves of the pumpkin, burnt on a bright fire, will cause flies to leave an apartment, or will destroy them instantly. Birds and other pets, as well as persons, should be removed from the room previous to the fumigation.

---

CEMENT FOR PICKLE AND PRESERVE JARS.—To two ounces of the best gum tragacanth, add six gills of cold water, and three grains of corrosive sublimate. Set it in a warm place for two or three days, frequently stirring it to assist the solution. This will be found to be a very useful paste for labels, or for pasting paper over jars holding pickles, preserves, fruits, &c., providing it does not come in contact with the condiment, to avoid which the jars should be first covered with paper, and then with the pasted paper.

---

TOMATO VINEGAR.—Take one bushel of ripe tomatoes, mash them in a tub, add one quart of molasses, and thoroughly mix the whole together. Let the tub stand several days, frequently stirring the mixture. When a decided vinegar odor is given off, the juice should be strained from the pomace and put into a cask. Vinegar so made is equal to the best.

---

WATERPROOF PAPER.—Housekeepers will be glad to know that the paper which they cover over fruit jars, can be made perfectly air and water proof, by brushing it over with boiled linseed oil and suspending it on a line, in the sun, until dry.

---

### JAPANESE DOMESTIC LIFE.

M. Humbert, the Swiss Minister at Jeddo, has lately published some amusing details of the domestic life of the Japanese. In Japan, marriage is the universal habit. Almost the only exceptions are to be found in the case of certain monastic orders, and among the ladies in attendance upon the Empress. Men marry at about twenty, and women at fifteen years of age, but except in the Buddhist sects, the act is marked by no religious ceremony. Among the presents displayed is always to be seen a double-lipped vase. At a given moment one of the bridesmaids advances, fills it with *saki*, and presents it alternately to the bridegroom and bride, until the goblet is emptied. Under this symbol the idea is conveyed that together the husband and wife must drink the cup of conjugal life to the dregs—whether it be filled with ambrosia or with gall. Japanese mothers have greater authority over their children than their fathers, and the rights of woman are so far recognized in the country that a woman has wielded the sceptre of the Mikados. But to return to the home life. The law of the country insists that each child shall be daily exposed to the air without clothes and with its head shaved, and in spite of both rain and sun. During infancy the child's ordinary playmates are a fat, short-legged dog, and fatter tailless cat. Instruction is never forced upon either parents or children; it is supposed to recommend itself naturally by its own intrinsic merits; and every man and woman throughout the empire is able to read, write, or cipher. The thirteenth day after birth every citizen receives his first name; on attaining his majority he takes a second; a third on his marriage; a fourth on being invested with any public function, which he changes upon attaining each higher grade, and so on to the name given to him after his death. The last is engraved on his tomb, and he is known by it to all succeeding generations.

